# Kindergarten children's attachment security, inhibitory control, and the internalization of rules of conduct

**DOI:** 10.3389/fpsyg.2013.00133

**Published:** 2013-03-27

**Authors:** Tobias Heikamp, Gisela Trommsdorff, Michel D. Druey, Ronald Hübner, Antje von Suchodoletz

**Affiliations:** ^1^Department of Psychology, University of KonstanzKonstanz, Germany; ^2^Department of Psychology, University of ZurichZurich, Switzerland; ^3^Department of Economics and Behavioral Sciences, University of FreiburgFreiburg, Germany

**Keywords:** attachment, inhibitory control, internalization, self-regulation, Stop-task, kindergarten children

## Abstract

Starting from research on relations between attachment and the development of self-regulation, the present study aimed to investigate research questions on relations among inhibitory control, internalization of rules of conduct (i.e., behavior regulation, concern occasioned by others transgressions, confession, reparation after wrongdoing), and attachment security. Attachment security and internalization of rules of conduct of German kindergarten children (*N* = 82) were assessed by maternal reports. Children's inhibitory control was measured with the Stop-task. Regression analyses revealed that inhibitory control was positively related to attachment security and to internalization of rules of conduct. Mediational analysis using a bootstrapping approach indicated an indirect effect of attachment security on internalization processes via inhibitory control. Implications for further research on the development of inhibitory control and internalization of rules of conduct are discussed.

In everyday routines, children have to suppress undesirable behaviors in order to follow their caregivers' requests (Kopp, 1982). The preschool period is marked by an increase in the ability of inhibitory control and children's motivation and ability to comply with rules of conduct (e.g., Kopp, 1982; Kochanska et al., 1994; Carlson, 2005). Inhibiting dominant responses is an important aspect of self-regulation because of its relation to children's internalization of rules of conduct, confession, reparation after wrongdoing, and sensitivity to rule violations committed by others (Kochanska et al., 1994, 1996, 1997; Hoffman, 2000; Eisenberg et al., 2011). However, significant individual differences in inhibitory control and internalization exist at preschool age (Kochanska et al., 1997; Blair and Diamond, 2008). From a developmental perspective, attachment theory may provide a framework for explaining individual differences. Past research found positive and direct relations between children's attachment security and emerging internalization of rules of conduct. For instance, previous studies showed that securely attached infants complied with maternal requests more often than insecurely attached infants (e.g., Stayton et al., 1973; Londerville and Main, 1981). Further research revealed that mothers of securely attached children create a context of reciprocity based on positive affect and emotional understanding which fosters children's willingness to internalize rules of conduct (Laible and Thompson, 2000, 2002; Van IJzendoorn and Sagi, 2008; for a review see Thompson et al., 2006). Other work, however, has suggested indirect effects of attachment security on children's internalization via children's self-regulation (e.g., Sroufe, 2005). Therefore, in the present study, we aimed to contribute to prior research by examining whether inhibitory control, as one aspect of self-regulation, mediates the relation between attachment security and internalization of rules of conduct.

Development of self-regulation and related internalization processes take place in cultural contexts in which different cultural values and norms prevail that children are expected to internalize (Trommsdorff, 2012). For instance, cross-cultural research has indicated cultural differences with regard to parents' socialization goals (e.g., obedience, self-control) and children's motivation and ability to inhibit behavior (e.g., Keller et al., 2006; Rubin et al., 2006). However, research on relations among attachment security, inhibitory control, and internalization processes conducted with European samples (e.g., Germany) is scarce. Therefore, we investigated the internalization of rules of conduct in a German sample of kindergarten children, choosing a different cultural context to gain first insight on cultural similarities and differences regarding these relations. Before we report our results, however, we provide a brief review of developmental literature on inhibitory control and the internalization of rules of conduct.

## Inhibitory control and internalization of rules of conduct

In the study of inhibitory control, different theoretical perspectives and methodological approaches co-exist. In developmental psychology, the concept of inhibition has often been related to temperament-based individual differences in self-regulation (e.g., effortful control) and their role for social and emotional development (Rothbart and Bates, 2006). Effortful control as a dimension of temperament has been defined more broadly as the competence to inhibit a dominant response and/or to activate a subdominant response, as well as to plan, and to detect errors (Rothbart and Bates, 2006; Eisenberg et al., 2011). In other areas such as in cognitive psychology, however, inhibition has been studied within the framework of executive functions (Zhou et al., 2012; Diamond, 2013). In the present study, we focus on inhibitory control, an executive function that serves to regulate behavior (Miyake et al., 2000) by suppressing dominant but inappropriate responses (e.g., Logan, 1994; see Friedman and Miyake, 2004 for an overview).

In past research, inhibitory control was positively associated with children's internalization of rules of conduct. In a cross-sectional study, children's inhibitory control, assessed by maternal reports, was positively associated with mothers' evaluations of their children's internalization of rules of conduct (Kochanska et al., 1994). Further longitudinal studies (Kochanska et al., 1996, 1997) relying on behavioral observations revealed positive relations between measures of inhibitory control (e.g., waiting for a snack) and children's internalization of rules of conduct (e.g., being alone with prohibited objects). Moreover, children high in inhibitory control were more likely to show other-oriented concern (Valiente et al., 2004), were more concerned about their own wrongdoing (Rothbart et al., 1994), and exhibited higher levels of prosocial behavior (Eisenberg et al., 1997, 2007).

Although the motivation and ability to follow rules of conduct, confession, reparation after wrongdoing, and sensitivity to rule violations committed by others are interrelated aspects of children's “active moral regulation” (Kochanska et al., 1994), the extent to which inhibitory control is related to each of these facets of internalization has not been investigated yet. Moreover, different developmental pathways underlying internalization, however, suggest different relations between inhibitory control and aspects of the internalization of rules of conduct. Children between 2 and 3 years of age, for instance, develop an understanding of social norms and show sensitivity to rule violations (Rakoczy and Schmidt, 2013). However, children at this age are still prone to impulsive reactions (e.g., to resist a temptation; Metcalfe and Mischel, 1999) that interfere with the motivation to follow rules of conduct (Kopp, 1982). After the age of 4, children are better able to successfully achieve a goal (e.g., to resist the temptation for an immediate reward; Mischel and Ayduk, 2011). This coincides with a significant increase of the motivation and ability to inhibit dominant responses between 3 and 5 years of age (Kopp, 1982; Carlson, 2005; Garon et al., 2008). These findings suggest that inhibitory control is more strongly associated with the ability to follow rules of conduct than with offering reparation after wrongdoing. Therefore, it is particularly informative to investigate relations between inhibitory control and different indicators of internalization separately.

## Attachment, inhibitory control, and internalization of rules of conduct

Previous studies revealed that attachment security was positively related to children's motivation and ability to comply with parental requests during mother-child-interactions in infancy (Stayton et al., 1973; Londerville and Main, 1981). However, empirical evidence for relations between attachment security and children's internalization of rules of conduct at kindergarten age is less clear. Laible and Thompson (2000) reported that attachment security showed a positive and significant association to mothers' reports of children's motivation and ability to follow rules of conduct in a sample of US-American children at the age of 4 years. However, the relation between attachment security and behavioral measures of children's motivation and ability to resist a temptation (i.e., to follow mothers' instructions not to play with attractive toys during their absence) was only marginally significant. In contrast to these results, Kochanska et al. (2004) did not find a significant relation between US-American children's attachment security, assessed at 14 months in the Strange Situation, and children's internalization of rules of conduct at the age of 6.

Although not yet investigated, previous studies suggest indirect effects of attachment security on children's internalization of rules of conduct. Given that recent research has suggested a direct relation between attachment security and inhibitory control, one possible pathway might be an indirect association of attachment security with internalization of rules of conduct via inhibitory control. For example, in a study by Booth-LaForce and Oxford (2008), attachment security longitudinally predicted mothers' ratings of children's motivation and ability to inhibit a dominant response. Moreover, delay of gratification as an early marker of children's motivation and ability to inhibit behavioral impulses (Eigsti et al., 2006) is positively related to attachment security (Jacobsen et al., 1997; Sethi et al., 2000). More recently, Bernier et al. (2012) have reported in a longitudinal study that children's attachment security assessed at age 2 was positively related to children's performance in executive function tasks measuring working memory, inhibitory control, and set shifting at the age of 3. Most notably, attachment security explained additional variance in children's executive functioning beyond that explained by parenting behavior, children's executive functioning at age 2 and other variables (e.g., socio-economic status, verbal ability). Thus, attachment security may be associated with children's internalization of rules of conduct because of the positive relation between attachment security and inhibitory control.

## Measuring inhibitory control

In past research, different methods have been used to assess inhibitory control at preschool age. These methods included questionnaires (e.g., parents' ratings; Kochanska et al., 1994) and observational measures (e.g., Kochanska et al., 1996, 1997). However, Else-Quest et al. (2006) concluded from the results of a meta-analytic study that there is an overall greater reliance on questionnaire data in the field of temperament research (e.g., concerning the assessment of inhibitory control and related concepts). In line with previous studies (e.g., Laible and Thompson, 2000), we used well validated questionnaires to assess children's attachment security and internalization of rules of conduct. Further, we decided to use an independent data source (i.e., behavioral measure) for the assessment of inhibitory control. A literature review revealed that a great variety of observational measures (e.g., test batteries including delaying, suppressing activity to a signal; for overviews see Spinrad et al., 2007; Garon et al., 2008) has been used to assess inhibitory control in the field of development psychology. Some of these measures, however, have been criticized for their poor construct validity and for being rather unspecific (Schachar and Logan, 1990; Oosterlaan et al., 1998). We therefore applied the Stop-task (Logan, 1994), which is an established procedure in cognitive psychology for specifically assessing inhibitory control. As the Stop-task has also been successfully applied in previous studies with children (e.g., Schachar and Logan, 1990; Carter et al., 2003) we chose this task in order to investigate the relations between inhibitory control, attachment security, and internalization of rules of conduct in the present study.

## The current study

The present study investigated relations between attachment security, inhibitory control and internalization of rules of conduct in a sample of German kindergarten children. Previous studies suggested direct and positive associations of inhibitory control with the internalization of rules of conduct and attachment security. Further evidence speaks to a direct and positive relation between attachment security and the internalization of rules of conduct. In extension of past research, the major aim within the current study was to investigate whether this relation is mediated by inhibitory control. In line with previous findings (e.g., Laible and Thompson, 2002), we therefore expected positive relations between attachment security and internalization of rules of conduct. Although research on attachment-related differences in inhibitory control is scarce (cf. Booth-LaForce and Oxford, 2008; Bernier et al., 2012), we hypothesized that inhibitory control would be positively associated with attachment security. Moreover, we assumed that inhibitory control is positively related to internalization of rules of conduct (Kochanska et al., 1996, 1997). We examined these relations separately for each aspect of internalization of rules of conduct (i.e., behavior regulation, concern occasioned by others' transgressions, confession, reparation) in order to further corroborate and extend the scope of previous studies.

## Methods

### Participants

In this study, 82 German kindergarten children (36 girls and 46 boys) and their mothers participated. The children were between 4.41 and 6.48 years old (*M* = 5.49; *SD* = 0.51). Mothers' education level was high. Most of the mothers (73.20%) had completed at least the first stage of tertiary education (i.e., BA or MA; Organization for Economic Co-operation and Development, 1999). Mothers rated their socioeconomic status on a 5-point scale (1 = low to 5 = upper). The sample was relatively homogeneous with regard to mothers' reported SES (*M* = 3.14; *SD* = 0.70). For their participation the mothers received a book coupon (value 5 €) and the children were allowed to choose a small toy at the end of each visit.

### Instruments and procedures

The mothers and their children visited the laboratory twice because this study was part of a larger project on children's self-regulation. During the first visit, the mothers answered questionnaires. During the second visit, children's inhibitory control was assessed.

#### Attachment security

Mothers evaluated their children's attachment behavior on a 9-point-scale using a German version of the Attachment Q-Sort (AQS, Waters and Deane, 1984). The AQS consists of 90 individual statements that are descriptive of the secure-base behavior of children. They were instructed to sort 10 items in each of nine piles ranging from 9 (“most like my child”) to 1 (“very unlike my child”) in terms of their relevance to the child. Mothers completed the AQS at their homes and handed their sorts in approximately 2 weeks after their first visit. Van IJzendoorn et al. (2004) concluded from their results that the AQS is less valid when used by parents. However, Moss et al. (2006) found in their study partial support for the validity of maternal ratings of kindergarten children's attachment security. Despite its limitations, in previous studies on internalization and attachment the AQS has been reported to be a reliable and valid instrument for assessing attachment security based on parental sorts (e.g., Laible and Thompson, 1998, 2000).

Schölmerich and Leyendecker (1992) provided a German criterion sort for attachment security that was strongly correlated with the standard sort based on US expert ratings (see Schölmerich and Van Aken, 1996). In order to obtain a child's security score, mothers Q-Sort profiles were correlated with the German criterion sort. The criterion sort was constructed by having experts sort the items to describe the hypothetical most secure child. Individual sorts were correlated with the criterion sort, and *r* (with a theoretical range of security scores from −1.00 = insecurely to 1.00 = securely attached) was used as a similarity index. Resulting correlations were transformed using Fishers *r*-to-*z* transformation to adjust the distribution by converting Pearson's *r* to the normally distributed variable *z*.

#### Inhibitory control

Inhibitory control as assessed in the Stop-task depends on a race between the primary task response execution process (“go” process) and the inhibition process triggered by the stop signal (“stop” process). The faster the stop process, the less likely the go process wins the race, resulting in a higher probability of inhibition, and vice versa (Logan and Cowan, 1984; Logan, 1994). Most important, within the Stop-task one can account for individual differences in responding to the “go” signal. This is relevant as it is more difficult to inhibit fast responses than slow ones (Logan, 1994). Mean stop signal reaction time (MSSRT) was obtained from the Stop-task in order to assess inhibitory control. MSSRT is the estimated time taken to inhibit an ongoing response.

**Figure 1:**
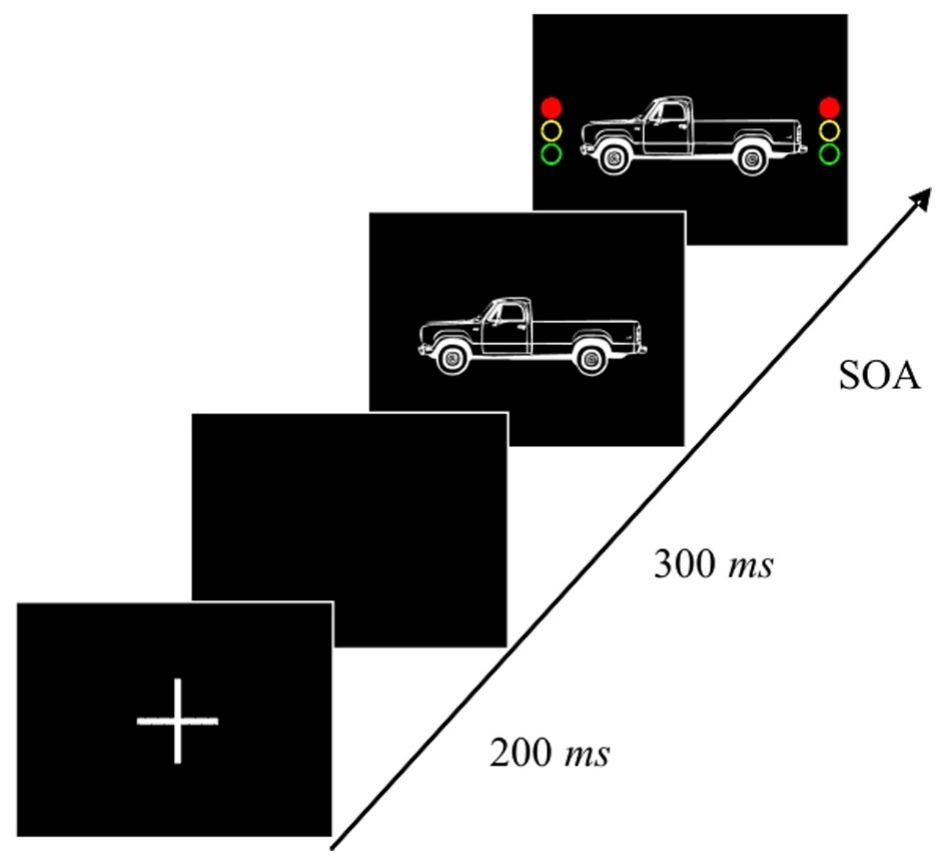
**Design of the Stop-task.** *Note*. Depicted is a stop-signal trial (1/3 of all trials in the test blocks). In the go-trials (all trials in the practice and baseline blocks, 2/3 of all trials in the test blocks), no stop signal appeared and the target stimulus disappeared either immediately after responding or after 2500 ms elapsed without a response being executed. Stimulus onset asynchrony (SOA) values were set as equally spaced proportions (i.e., 20, 40, 60, and 80%) of mean go reaction times from the baseline blocks (see text for further details).

***Apparatus, stimuli, and tasks*.** Stimulus presentation and response recording were controlled by an IBM-compatible PC. Pictures of cars facing either left or right served as primary task stimuli, and a red traffic light was presented as stop signal (see Figure 1). The primary task stimuli appeared always at the center of the screen and the stop signal was presented to the left and right of the primary task stimulus.

The children were instructed to press a left button with their index finger of the left hand upon the presentation of a car facing to the left, and to press a right button with their index finger of the right hand upon the presentation of a car facing to the right. Furthermore, they were instructed to respond upon the presentation of the cars as quickly and as accurately as possible, but to stop their responses if the traffic light appeared. They were told not to wait for the traffic light, as it would appear only occasionally. Instructions were given orally and the general procedure was illustrated using cards depicting the stimuli. Mean reaction times (RT) and error rates (ER) were checked after each block and the children received feedback about their performance. Children were handed coins after each block if they performed well according to the given instructions in order to ensure that they did not wait for the traffic light. Additionally, they were told that they can exchange the collected coins for a toy at the end of the experiment. The task, including instruction and training blocks, took about 45 min.

***Procedure and design*.** A given trial started with the presentation of a fixation cross for 200 ms, followed by a blank screen for 300 ms. The primary task stimulus was then presented and remained visible until a response was executed (on go-trials and on non-successful stop-trials). After response execution, a blank screen appeared and remained for 1000 ms, before the next trial started. On successful stop-trials and on go-trials on which erroneously no response was executed, the primary task stimulus disappeared after 2500 ms, followed also by a 1000 ms blank inter-trial interval.

In total, the children performed 14 blocks. In the first four blocks, no stop-signal appeared and each block contained 24 go trials. Of these four blocks, the first two blocks served as practice only and were not further analyzed. The following two blocks (3 and 4) then served as a baseline measure for computing the individual stop-signal delays for each child in the following Stop-task blocks. In the critical test blocks (5–14), stop-signals appeared on one third of the trials. Each of these blocks consisted of 36 trials (24 go-trials, 12 stop-trials). The stop signals were presented at four different Stimulus Onset Asynchronies (SOAs) after the onset of the primary task go stimulus (car). The SOAs were chosen such that the shortest would yield a probability of inhibition close to 1, whereas the longest would produce a probability of inhibition close to 0. Following Carter et al. (2003), the SOAs were set as equally spaced proportions (20, 40, 60, and 80%) of mean individual go-RT from blocks 3 and 4, in which no stop-signal appeared. Since it has been shown that the theoretically most relevant SOAs are those in the middle (i.e., 40 and 60%; see Logan, 1994), they appeared twice as often as the shortest (i.e., 20%) and the longest (i.e., 80%) SOAs.

The latency of the stop process, i.e., the MSSRT, was estimated separately for each child (see Logan, 1994). For further analyses, the resulting MSSRT variable was multiplied by −1. Thus, larger values indicated more efficient inhibitory control.

#### Internalization of rules of conduct

Mothers answered items of the “My Child” questionnaire (Kochanska et al., 1994) on a 7-point scale (1 = extremely untrue, not at all characteristic to 7 = extremely true, very characteristic) in order to assess children's internalization of rules of conduct. *Behavior Regulation* assesses children's motivation and ability to behave according to social standards in the absence of a caregiver (20 items; e.g., “Rarely repeats previously prohibited behavior even if adult is not present.”). *Concern Occasioned by Others' Transgressions* focuses on children's sensitivity for rule violations by other persons (7 items; e.g., “Gets upset when a guest breaks a household rule.”). *Confession* includes children's motivation and ability to admit their own wrongdoings (7 items; “Will spontaneously admit fault or wrongdoing.”) and *Reparation* denotes children's willingness to make amends for their misconduct (9 items; “Eager to make amends for doing something naughty.”). Cronbach's Alpha coefficients were 0.87 for *Behavior Regulation*, 0.83 for *Concern Occasioned by Others' Transgressions*, 0.72 for *Confession*, and 0.72 for *Reparation*. All scales had good internal consistencies, comparable to those reported by Kochanska et al. (1994).

### Data analysis

Separate regression analyses were computed in order to test direct relations between attachment security, inhibitory control, and internalization. In each model children's age and gender were entered in a first block because previous studies yielded significant associations of age and gender with inhibitory control and aspects of internalization (e.g., Kochanska et al., 1994, 1996; Bjorklund and Kipp, 1996). Socio-economic status served as a control variable as family demographics were related to development of executive functions and internalization in previous research (e.g., Groenendyk and Volling, 2007; Rhoades et al., 2011). Finally, a bootstrapping procedure was applied in order to test indirect effects of attachment security on internalization of rules of conduct via inhibitory control because it is recommended as the method of choice for analyses with small samples in child development research (Dearing and Hamilton, 2006). Preacher and Hayes (2004; see also Shrout and Bolger, 2002; MacKinnon et al., 2007) recommended bootstrapping procedures for small samples because it makes no assumptions with respect to normality. Point estimates and 95% confidence intervals were estimated for the indirect effects. Using the bootstrapping method, altogether 5000 random samples from the dataset were drawn.

## Results

**Figure 2:**
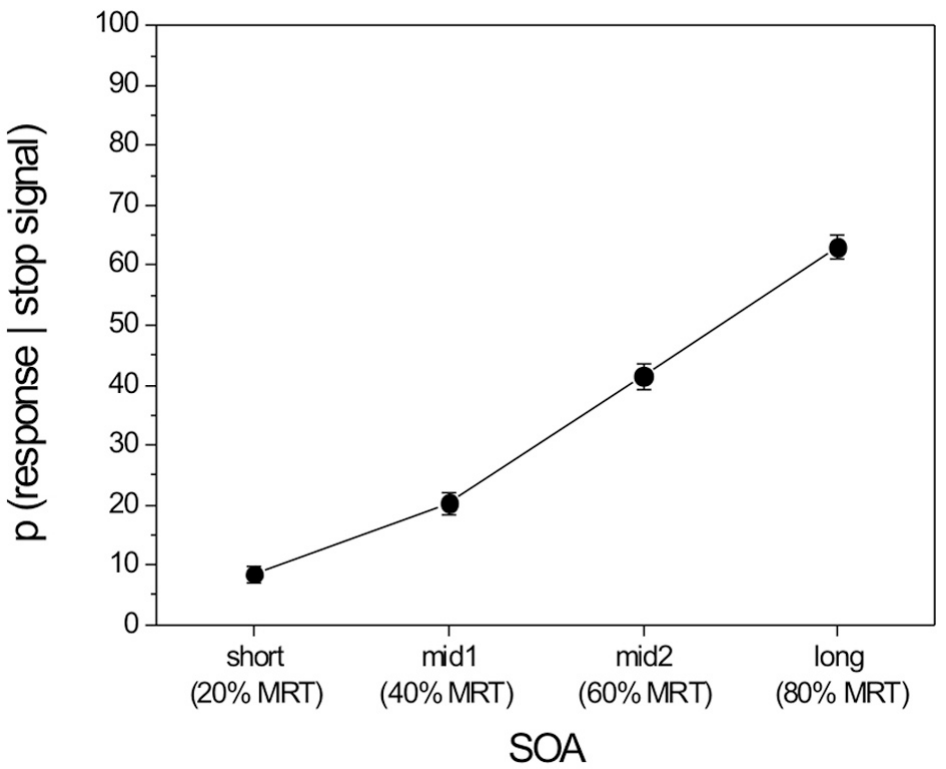
**Probability (in %) of responding to the go-stimulus despite that a stop signal appeared [*p*(response | stop signal)] at the individual SOAs.** *Note*. MRT, Mean Response Time in the baseline blocks. Error bars represent standard errors of the means.

The mean score for attachment security in the present study was somewhat higher than that for non-clinic samples (*M* = 0.32; *SD* = 0.16) reported in a meta-analysis by Van IJzendoorn et al. (2004). The MSSRT was comparable to that in another study with a group of 6–8 years old children (Williams et al., 1999). All descriptive statistics are presented in Table 1. Figure 2 shows the probabilities of responding given a stop signal in the Stop-task depending on the SOA.

**Table 1:** **Descriptive statistics**.

	***M***	***SD***	**Range**
Attachment security	0.44	0.22	−0.11, 0.89
Inhibitory control^a^	−336.16	114.29	−917.75, −139.75
**INTERNALIZATION MEASURES**			
Behavior regulation	4.22	0.81	2.05, 6.05
Concern about transgressions	4.96	0.97	1.57, 6.86
Confession	4.59	0.93	2.14, 6.57
Reparation	5.12	0.81	2.33, 7.00

*Note*.

a *Inhibitory control was measured by Mean Stop Signal Reaction Time (MSSRT in ms) in the Stop-task. MSSRT is an estimate of the time taken to inhibit a response following the presentation of a stop signal. For these analyses the resulting MSSRT variable was multiplied by −1. Thus, a higher value indicates better inhibitory control*.

The mean values of the four internalization scales were similar to those found in other studies (e.g., Kochanska et al., 1994). Pearson correlations revealed that the four dimensions of internalization were positively and significantly related. Correlation coefficients ranged from 0.31 to for the relation between *Concern about transgression* and *Confession* to 0.53 for the relation between *Confession* and *Reparation*. Moreover, inhibitory control was significantly and positively related to attachment security, behavior regulation, and confession. Furthermore, correlation analyses revealed positive and significant relations of attachment security to behavior regulation, confession, and reparation (see Table 2).

**Table 2:** **Correlations between attachment security, inhibitory control, and internalization measures**.

	**1**	**2**	**3**	**4**	**5**	**6**
1. Attachment security	1					
2. Inhibitory control	0.27^*^	1				
**INTERNALIZATION MEASURES**						
3. Behavior regulation	0.38^**^	0.34^**^	1			
4. Concern about transgressions	0.21^+^	0.13	0.47^**^	1		
5. Confession	0.22^*^	0.25^*^	0.33^**^	0.31^**^	1	
6. Reparation	0.33^**^	0.22^+^	0.53^**^	0.53^**^	0.47^**^	1

+ p < 0.10,

* p < 0.05,

** *p < 0.01*.

### Direct links between attachment, inhibitory control, and internalization

To test relations between attachment security and internalization of rules of conduct separate regression analyses were computed with attachment security as predictor variable for *Behavior Regulation*, *Concern Occasioned by Others' Transgressions*, *Confession*, and *Reparation*. In block 1, child's age, gender, and mother's SES were entered in each of the regression analyses (see Table 3). SES was positively associated with *Behavior Regulation* (β = 0.26, *p* < 0.05), and girls showed a higher level of internalization in comparison to boys with respect to sensitivity to transgressions occasioned by others' (β = 0.32, *p* < 0.01).

**Table 3:** **Summary of regression analyzes to predict internalization measures**.

	**Step 1**			**Step 2**			***R*^2^**	**Δ*R*^2^**
	***B***	***SE B***	**β**	***B***	***SE B***	**β**		
**BEHAVIOR REGULATION**								
Step 1							0.11^*^	0.11^*^
Age	0.21	0.17	0.13	0.20	0.16	0.13		
Gender^a^	0.27	0.17	0.16	0.23	0.16	0.14		
Perceived SES	0.30	0.12	0.26^*^	0.26	0.12	0.22^*^		
Step 2							0.22^**^	0.11^**^
Attachment security				1.27	0.38	0.34^**^		
**CONCERN OCCASIONED BY OTHERS' TRANSGRESSION**								
Step 1							0.13^*^	0.13^*^
Age	−0.21	0.20	−0.11	−0.21	0.20	−0.11		
Gender^a^	0.61	0.21	0.32^**^	0.59	0.20	0.30^**^		
Perceived SES	0.18	0.15	0.13	0.15	0.15	0.11		
Step 2							0.16^**^	0.03^+^
Attachment security				0.81	0.47	0.18^+^		
**CONFESSION**								
Step 1							0.02	0.02
Age	0.15	0.21	0.08	0.15	0.20	0.08		
Gender^a^	−0.12	0.21	−0.06	−0.14	0.21	−0.08		
Perceived SES	0.09	0.15	0.07	0.06	0.15	0.05		
Step 2							0.06	0.05^+^
Attachment security				0.93	0.48	0.22^+^		
**REPARATION**								
Step 1							0.04	0.04
Age	−0.02	0.18	−0.01	−0.02	0.17	−0.02		
Gender^a^	0.12	0.18	0.07	0.09	0.17	0.05		
Perceived SES	0.21	0.13	0.18	0.17	0.12	0.14		
Step 2							0.13^*^	0.09^**^
Attachment security				1.14	0.40	0.31^**^		

*Note*.

a *Dummy coded: 0 = boys, 1 = girls*.

+ *p < 0.10*.

* *p < 0.05*.

** *p < 0.01*.

In block 2, attachment security was entered as a predictor. The results revealed positive associations of attachment security with *Behavior Regulation* (β = 0.34, *p* < 0.01) and *Reparation* (β = 0.31, *p* < 0.01). The relation between attachment security and *Concern Occasioned by Others' Transgressions* (β = 0.18, *p* = 0.09) and the relation between attachment security and *Confession* (β = 0.22, *p* = 0.06) were marginally significant. The variables included in each of the models explained between 6% (*Confession*) and 22% (*Behavior Regulation*) of the variability of each dimension.

A second regression model tested whether attachment security was related to inhibitory control (see Table 4). Inhibitory control was positively related to child's age (β = 0.27, *p* < 0.05) and attachment security (β = 0.26, *p* < 0.05). The full model accounted for 18% of the total variance in inhibitory control.

**Table 4:** **Regression analysis to predict inhibitory control**.

	**Step 1**			**Step 2**			***R*^2^**	**Δ *R*^2^**
	***B***	***SE B***	**β**	***B***	***SE B***	**β**		
**INHIBITORY CONTROL**								
Step 1							0.11^*^	0.11^*^
Age	61.11	23.96	0.27^*^	60.50	23.23	0.27^*^		
Gender^a^	−15.40	24.48	−0.07	−18.98	23.78	−0.08		
Perceived SES	29.44	17.43	0.18^+^	24.70	17.01	0.15		
Step 2							0.18^**^	0.06^*^
Attachment security				133.54	54.53	0.26^*^		

*Note*.

a *Dummy coded: 0 = boys, 1 = girls*.

+ *p < 0.10*.

* *p < 0.05*.

** *p < 0.01*.

Moreover, regression analyses were computed to test for the relations between inhibitory control and the internalization of rules of conduct. Inhibitory control was positively associated with *Behavior Regulation* (β = 0.31, *p* < 0.01), *Confession* (β = 0.23, *p* = 0.05), *Reparation* (β = 0.22, *p* = 0.06) and *Concern Occasioned by Others' Transgressions* (β = 0.18, *p* = 0.10). The variables included in each of the models explained between 7% (*Confession*) and 19% (*Behavior Regulation*) of the variability of each dimension (see Table 5).

**Table 5:** **Multiple regression analyzes for inhibitory control predicting internalization measures**.

	**BEHAVIOR REGULATION**			**CONCERN OCCASIONED BY OTHERS' TRANSGRESSIONS**		
	***B***	***SE B***	**β**	***B***	***SE B***	**β**
Age	0.08	0.17	0.05	−0.30	0.21	−0.16
Gender^a^	0.30	0.17	0.18^+^	0.64	0.20	0.33^**^
Perceived SES	0.24	0.12	0.21^+^	0.13	0.15	0.10
Inhibitory control	0.00	0.00	0.31^**^	0.00	0.00	0.18
	**CONFESSION**			**REPARATION**		
	***B***	***SE B***	**β**	***B***	***SE B***	**β**
Age	0.04	0.21	0.02	−0.11	0.18	−0.07
Gender^a^	−0.09	0.21	−0.05	0.14	0.18	0.09
Perceived SES	0.04	0.15	0.03	0.16	0.13	0.14
Inhibitory control	0.00	0.00	0.23^+^	0.00	0.00	0.22^+^

*Note. R^2^ of the full model was 0.19 (p < 0.01) for Behavior regulation, 0.16 (p < 0.01) for Concern occasioned by others' transgressions, 0.07 (p = 0.27) for Confession, and 0.08 (p = 0.16) for Reparation. Change in R^2^(Δ R^2^) when including the variable Inhibitory control in the model was 0.08 (p < 0.01) for Behavior regulation, 0.03 (p = 0.10) for Concern occasioned by others' transgressions, 0.05 (p = 0.05) for Confession, and 0.04 (p = 0.06) for Reparation*.

a *Dummy coded: 0 = boys, 1 = girls*.

+ *p < 0.10*.

** *p < 0.01*.

### Indirect effects of attachment on internalization through inhibitory control

**Figure 3:**
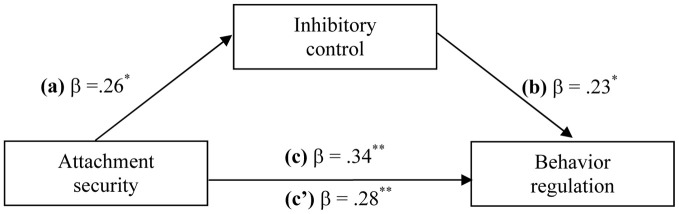
**Mediation model for the relations between attachment security, inhibitory control, and behavior regulation.** Path estimates for the direct effect of attachment security on inhibitory control **(a)**, the direct effect of inhibitory control on behavior regulation **(b)**, the direct **(c)**, and indirect effect **(c')** of attachment security on behavior regulation (controlling for socio-economic status, child's age, and gender). Standardized regression coefficients are presented.

As *Behavior Regulation* was the only internalization measure that was significantly related to inhibitory control a simple mediation model using a bootstrapping procedure (Preacher and Hayes, 2004, 2008) was computed with attachment security as predictor, inhibitory control as the mediator and *Behavior Regulation* as the outcome, including socioeconomic status, children's age and gender as control variables (see Figure 3).

The indirect effect of attachment security on *Behavior Regulation* was significant for inhibitory control with a point estimate of 0.2273 and a 95% bootstrap confidence interval of 0.0426–0.5924. This result indicates an indirect effect of attachment security on *Behavior Regulation* through inhibitory control because the confidence interval did not include zero (Preacher and Hayes, 2008).

Following the recommendations by Fairchild et al. (2009) an *R*^2^ effect size was computed for the significant indirect effect. The resulting *R*^2^_med_ value of 0.05 indicated that about 5% of the variance in *Behavior Regulation* can be attributed to the indirect effect of attachment security through inhibitory control. Considering that attachment security and inhibitory control explained overall 20% of the variance in children's *Behavior Regulation* we can conclude that a proportion of 25% (0.05/0.20) of the explained variance in the model was due to the indirect effect.

## Discussion

The present study corroborated and extended previous findings on relations among attachment security, inhibitory control, and internalization of rules of conduct. In line with previous results from studies with US-American children (e.g., Laible and Thompson, 2000), mothers' reports revealed that securely attached children showed higher compliance with rules of conduct than insecurely attached children. Extending findings from previous studies, the present study has revealed positive relations between children's attachment security and children's motivation and ability to offer compensation after their misconduct. Furthermore, consistent with other studies (Kochanska et al., 1994, 1996, 1997; Booth-LaForce and Oxford, 2008), the present results revealed significant and positive relations of inhibitory control to internalization of rules of conduct and attachment security. Although little research has investigated direct relations between attachment security and inhibitory control (cf. Booth-LaForce and Oxford, 2008; Bernier et al., 2012), the findings from our study are in line with growing evidence that attachment security plays an important role for the development of individual differences with regard to different aspects of self-regulation (Sroufe, 2005) and executive functions (Bernier et al., 2012), including also inhibitory control (Schore, 2000). In extension of previous studies, the present study revealed an indirect effect of attachment security on internalization of rules of conduct through inhibitory control. Attachment security and inhibitory control accounted each for a significant amount of variance in behavior regulation above and beyond the variance that was explained by children's age, gender, and SES. The proportion of variance in behavior regulation explained by attachment security (11%) and inhibitory control (8%) was somewhat higher than the amount of variance attachment security accounted for in inhibitory control (6%). Although the present study revealed statistically significant effects of attachment security and inhibitory control on internalization, overall the effect sizes were lower than those reported in previous studies (e.g., Kochanska et al., 1997; Laible and Thompson, 2000; Bernier et al., 2012). Past research suggested that internalization of rules of conduct is associated with attachment security (e.g., Laible and Thompson, 2000) and inhibitory control (e.g., Kochanska et al., 1996). However, the present study has revealed only significant indirect effects of attachment on children's behavior regulation. Confession or reparation usually follows children's wrongdoing. Complex cognitive and emotional processes (e.g., perspective taking, guilt) underlie the motivation to compensate for wrongdoing (e.g., Tangney et al., 2007). In contrast, behavior regulation is typically reflected more directly in children's actions, such as refraining from prohibited activities. Future studies may further address the question of whether specific associations of inhibitory control to single aspects of internalization exist. Alternatively, specific relations between inhibitory control and aspects of internalization (e.g., behavior regulation) may be explained by a common factor underlying children's internalization that is associated with inhibitory control, as the aspects of internalization are significantly interrelated. Moreover, further research should investigate whether the strength of relations between inhibitory control and the facets of internalization is stable over time or changes through the course of development.

Although it is an interesting finding that inhibitory control mediated the relation between attachment security and internalization of rules of conduct, we are not able to draw any conclusions regarding causality. Further research is necessary in order to investigate whether attachment experiences influence inhibitory control and internalization longitudinally or concurrently. We may only speculate whether links between attachment security, inhibitory control, and internalization of rules of conduct are direct or indirect in nature. From a longitudinal perspective, a stress buffering effect of early attachment experiences (Schieche and Spangler, 2005) may be related to the development of inhibitory control during childhood (Blair and Ursache, 2011). Furthermore, inhibitory control may facilitate appropriate regulation of behavior in disciplining contexts (e.g., attention to parental messages) and therefore promotes understanding and acceptance of maternal messages (Grusec and Davidov, 2007). However, reciprocal influences of a child's self-regulation capacities and maternal parenting should also be considered in future studies (Dennis, 2006; Trommsdorff and Cole, 2011).

The results of the present study support past findings that revealed positive associations of internalization of rules of conduct with inhibitory control and attachment security in samples of US-American children (e.g., Kochanska et al., 1996, 1997; Laible and Thompson, 2000). However, a recent study revealed substantial cultural differences with regard to the degree to which conformity with social rules is expected in a cultural context (Gelfand et al., 2011). As previous research indicated cultural differences in the development of inhibitory control (e.g., Sabbagh et al., 2006) and in the meaning of child compliance (e.g., Chen et al., 2003) it is important to study the function of inhibitory control for children's development of self-regulation systematically in different cultural contexts. For instance, future research should investigate the relative contribution of different mechanisms (e.g., emotional understanding, inhibitory control) on the internalization of rules of conduct in different cultural contexts. In socialization contexts in which obedience is highly emphasized (e.g., Japan) contexts of reciprocal parent-child interactions that promote emotional understanding and foster internalization are less likely to occur than in cultures in which independence-oriented parenting patterns prevail (e.g., Germany; see Trommsdorff and Kornadt, 2003). Moreover, in sociocultural contexts that emphasize obedience and self-control, but focus less on individual needs, inhibitory control develops earlier than in independence-oriented socialization contexts (e.g., Chen et al., 2003; Sabbagh et al., 2006). This could mean that inhibitory control may be more strongly associated with internalization processes in cultures that emphasize socialization goals related to interdependence, conformity, and obedience.

A potential methodological limitation in the present study is that the AQS was completed by the mothers and not by trained external observers (Van IJzendoorn et al., 2004). Although maternal ratings tend to be influenced by child's temperament, mothers' reports provide important information on their attachment relationships with their children because they have the best access to a representative sample of their children's behavior (Tarabulsy et al., 2008). Based on our data, however, no conclusions can be drawn as to how differences in self-regulation were associated with different insecure (i.e., insecure-avoidant; insecure-ambivalent) attachment patterns. A further limitation is that children's attachment security and internalization of rules of conduct were both assessed by maternal reports. To minimize eventually shared method variance, the administration of maternal reports was separated in time. Shared method variance is sometimes seen as a limitation with regard to the interpretation of significant correlations. However, there is growing evidence questioning the assumption that shared sources of measurement lead to common method variance and a problem regarding a study's validity because of an artificial inflation of correlation coefficients (e.g., Spector, 2006). Indeed, zero-order correlations between attachment security and some aspects of internalization were rather weak and non-significant. Using different data sources (e.g., teachers' and parents' reports), though, is also not without problems because cross-informant agreement for ratings of children's social competence is rather low suggesting that the magnitude of correlations can vary simply depending on the informants who evaluate children's behavior [see e.g., the meta-analytic study by Renk and Phares (2004)].

In the present study, we used a behavioral measure (i.e., Stop-task) in order to have a further data source available for the assessment of inhibitory control. As the majority of studies in the field of temperament research (e.g., effortful control) are based on questionnaire data [see e.g., the meta-analytic study by Else-Quest et al. (2006)] this is an important extension of past research. Although Diamond (2013) considered the Stop-task as a rather artificial instrument for measuring inhibition, the present study showed that it is nevertheless significantly related with mothers' reports of children's attachment security and internalization of rules of conduct. Thus, contrary to this perspective of Diamond (2013), we consider it as strength of our study that we have used the Stop-task to assess inhibitory control. The Stop-task is an established instrument in cognitive psychology, taking limitations of previously used measures for inhibitory control into account (Schachar and Logan, 1990; Oosterlaan et al., 1998). For instance, the Stop-task takes individual differences in general response speed into account. This is particularly important because it is more difficult to inhibit fast responses than slower ones. Research in kindergarten children on relations between Stop-task based inhibitory control measures and children's social functioning is scarce. However, inhibitory control measures derived from the Stop-task have also been criticized. Children with ADHD, for instance, tend to exhibit slower RTs to Go-stimuli than healthy controls, which affect the estimated SSRT (Castellanos et al., 2006). Fortunately, this deficit is relevant only when comparing different samples of children (e.g., in a control group design) and, therefore, does not apply to the present study. Nevertheless, it would be desirable to use several different methods and data sources (e.g., parents' reports, observational measures; see e.g., Weisz et al., 1995) in future studies in order to rule out eventual common method biases and to further corroborate the present findings.

## Conclusions

Starting from research on relations between attachment security and internalization processes, the present findings clearly attested to positive relations of internalization of rules of conduct to attachment security and children's motivation and ability to inhibit a dominant response. Extending previous findings, inhibitory control was assessed in a German sample of kindergarten children with the Stop-task. The present results have revealed that the Stop-task is a valid measure that is positively related to mothers' reports of children's attachment security and internalization of rules of conduct. The indirect effect of attachment security on internalization of rules of conduct via inhibitory control is a new finding that contributes to a better understanding of children's internalization of rules of conduct. Further research is necessary in order to study the underlying developmental mechanisms for these effects.

### Conflict of interest statement

The authors declare that the research was conducted in the absence of any commercial or financial relationships that could be construed as a potential conflict of interest.
